# Pan-genomic and transcriptomic analyses of *Leuconostoc mesenteroides* provide insights into its genomic and metabolic features and roles in kimchi fermentation

**DOI:** 10.1038/s41598-017-12016-z

**Published:** 2017-09-14

**Authors:** Byung Hee Chun, Kyung Hyun Kim, Hye Hee Jeon, Se Hee Lee, Che Ok Jeon

**Affiliations:** 1 Department of Life Science, Chung-Ang University, Seoul, 06974 Republic of Korea; 2 Microbiology and Functionality Research Group, World Institute of Kimchi, Gwangju, 61755 Republic of Korea

## Abstract

The genomic and metabolic features of *Leuconostoc* (*Leu*) *mesenteroides* were investigated through pan-genomic and transcriptomic analyses. Relatedness analysis of 17 *Leu*. *mesenteroides* strains available in GenBank based on 16S rRNA gene sequence, average nucleotide identity, *in silico* DNA-DNA hybridization, molecular phenotype, and core-genome indicated that *Leu*. *mesenteroides* has been separated into different phylogenetic lineages. Pan-genome of *Leu*. *mesenteroides* strains, consisting of 999 genes in core-genome, 1,432 genes in accessory-genome, and 754 genes in unique genome, and their COG and KEGG analyses showed that *Leu*. *mesenteroides* harbors strain-specifically diverse metabolisms, probably representing high evolutionary genome changes. The reconstruction of fermentative metabolic pathways for *Leu*. *mesenteroides* strains showed that *Leu*. *mesenteroides* produces various metabolites such as lactate, ethanol, acetate, CO_2_, mannitol, diacetyl, acetoin, and 2,3-butanediol through an obligate heterolactic fermentation from various carbohydrates. Fermentative metabolic features of *Leu*. *mesenteroides* during kimchi fermentation were investigated through transcriptional analyses for the KEGG pathways and reconstructed metabolic pathways of *Leu*. *mesenteroides* using kimchi metatranscriptomic data. This was the first study to investigate the genomic and metabolic features of *Leu*. *mesenteroides* through pan-genomic and metatranscriptomic analyses, and may provide insights into its genomic and metabolic features and a better understanding of kimchi fermentations by *Leu*. *mesenteroides*.

## Introduction

*Leuconostoc* (*Leu*.) *mesenteroides* comprises Gram-positive, catalase-negative, facultatively anaerobic, non-spore-forming, and spherical heterofermentative and mostly dextran-producing lactic acid bacteria (LAB), with coccus shapes and relatively low G + C contents^1, 2^. *Leu*. *mesenteroides* members are reported to be mainly responsible for the fermentation of various vegetables, such as kimchi (a Korean fermented vegetable food) and sauerkraut (pickled cabbage), under low temperature and moderate salinity conditions, although some *Leu*. *mesenteroides* strains have been isolated from dairy products such as cheese^2–6^. In particular, *Leu*. *mesenteroides* strains were found to be the major LAB, along with *Lactobacillus* (*L*.) *sakei* and *Weissella koreensis*, present during kimchi fermentation, suggesting that they are well adapted to kimchi fermentation conditions^3, 7, 8^. Moreover, because *Leu*. *mesenteroides* strains produce mannitol, a compound with antidiabetic and anticarcinogenic properties known for imparting a refreshing taste, and bacteriocins during fermentation and have some health improving effects^3, 9, 10^, they have been considered as starter cultures for kimchi fermentation or potential probiotics in industries^11–13^.

Although *Leu*. *mesenteroides* strains are generally considered to be non-infectious agents in humans, there have been some clinical reports that *Leu*. *mesenteroides* might be associated with certain human diseases such as brain abscess, endocarditis, nosocomial outbreaks, and central nervous system tuberculosis^14–17^. In addition, there is a report that *Leu*. *mesenteroides* can cause spoilage in some types of food products^18^. These reports suggest that further studies on the physiological and fermentative properties of *Leu*. *mesenteroides* strains are needed to vouch for the safety and quality of kimchi and sauerkraut products fermented with *Leu*. *mesenteroides*.

The 16S rRNA gene sequences have been widely used for the identification and diversity analysis of bacterial species. However, they are not appropriate for bacteria with high 16S rRNA gene sequence similarities such as LAB, suggesting that numerous bacterial strains that have been described as members of *Leu*. *mesenteroides* in previous studies, including the previously mentioned clinical reports, may not belong to *Leu*. *mesenteroides*. For example, *Leu*. *mesenteroides* ssp. *suionicum* was originally a subspecies member of *Leu*. *mesenteroides* due to high 16S rRNA gene sequence similarities (>99.72%), but was reclassified as a new species of the genus *Leuconostoc* (*Leu*. *suionicum*) based on genome comparisons^2, 19^. With the development of high-throughput and low-cost sequencing technologies, genomic information-based approaches have been extensively considered for the comprehensive understanding of metabolic properties and lifestyle traits of a microorganism. In particular, with the recent explosive increase of genome sequencing data, the concept of pan-genome has been introduced to explore the genomic and metabolic diversity of a given phylogenetic clade^20–23^. Because a pan-genome describes the entire genomic repertoire, representing all possible metabolic and physiological properties of a given phylogenetic clade and encodes for all possible lifestyles of a bacterial species, a pan-genome analysis provides insights into the genomic and metabolic features as well as a comprehensive understanding of the genome diversities and lifestyle traits of a bacterial species^24–29^. Therefore, in this study, we investigated the genome diversities and the genomic and metabolic features of *Leu*. *mesenteroides* strains using all genomes (pan-genome) of *Leu*. *mesenteroides* available in GenBank. In addition, we reconstructed the fermentative metabolic pathways of *Leu*. *mesenteroides* strains based on their pan-genome and examined their fermentative metabolic features during kimchi fermentation, through a transcriptomic analysis.

## Results and Discussion

### Relatedness of *Leu*. *mesenteroides* strains based on 16S rRNA gene sequences

**Figure 1:**
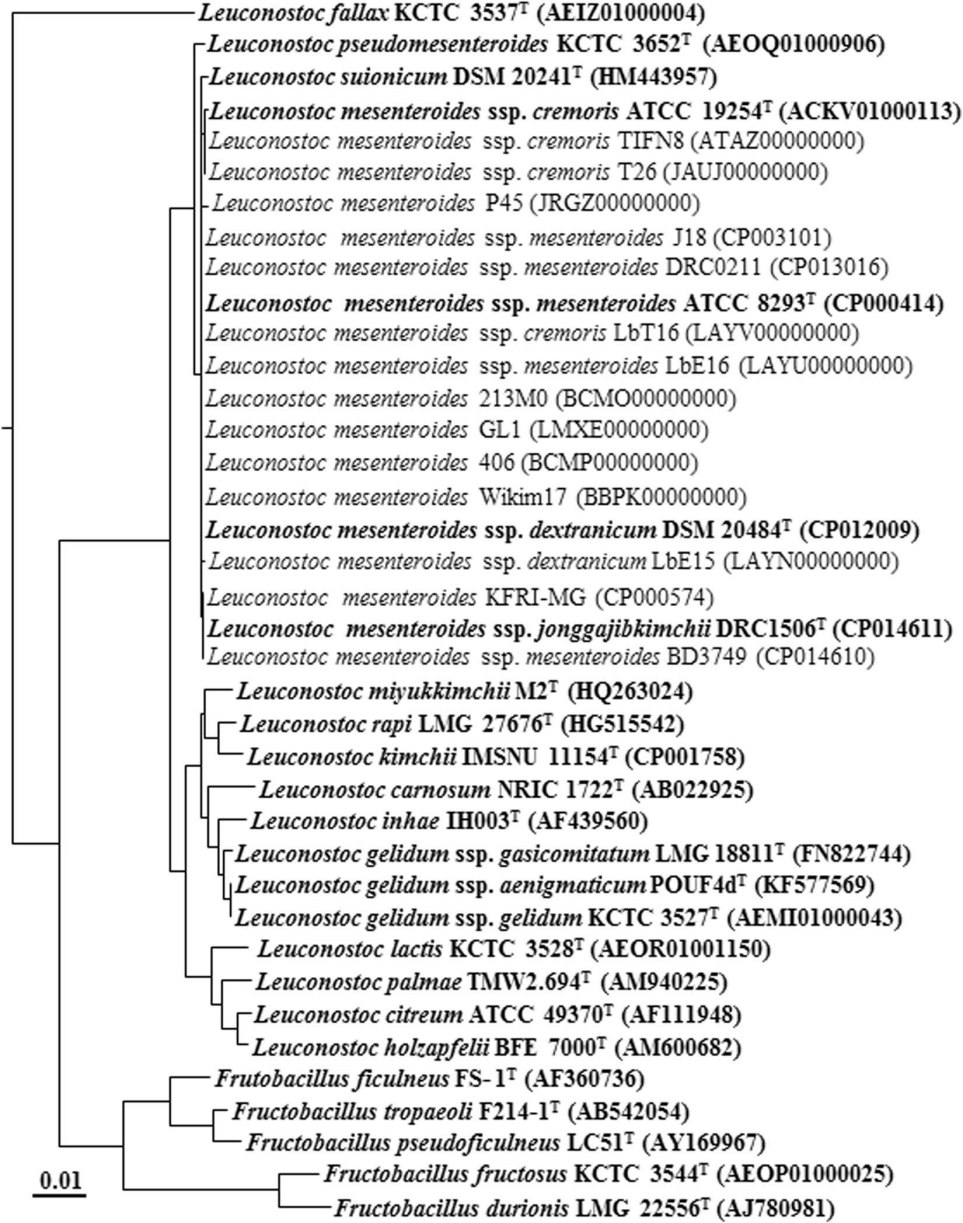
A phylogenetic tree using the NJ algorithm based on 16S ribosomal RNA sequences showing the phylogenetic relationships among *Leuconostoc mesenteroides* strains and related taxa. *Weissella viridescens* NRIC 1536^T^ was used as an outgroup (not shown). The type strains are highlighted in bold. The scale bar equals 0.01 changes per nucleotide.

The genomes of all *Leu*. *mesenteroides* strains available in GenBank and the type strains of closely related species, *Leu*. *suionicum* and *Leu*. *pseudomesenteroides*, which had more than 99.54% 16S rRNA gene sequence similarities, were retrieved and their general features are described in Table 1. To infer their phylogenetic relationships, a phylogenetic tree based on the 16S rRNA gene sequences was constructed with other closely related relatives (Fig. 1). All *Leu*. *mesenteroides* strains were clustered into a single phylogenetic lineage without any clear lineage differentiation. However, the phylogenetic tree showed that whereas *Leu*. *pseudomesenteroides* formed a distinct phylogenetic lineage from *Leu*. *mesenteroides* strains, *Leu*. *suionicum*, which was recently reclassified as a new species of the genus *Leuconostoc* from a subspecies of *Leu*. *mesenteroides* ^2, 19^, was not clearly separated from other *Leu*. *mesenteroides* strains by the 16S rRNA gene sequences. Currently, *Leu*. *mesenteroides* includes four type subspecies with valid published names: *Leu*. *mesenteroides* subsp. *mesenteroides*, *Leu*. *mesenteroides* subsp. *cremoris*, *Leu*. *mesenteroides* subsp. *dextranicum*, and *Leu*. *mesenteroides* subsp. *jonggajibkimchii* ^2^. However, the phylogenetic tree also showed that the four *Leu*. *mesenteroides* subspecies were not differentiated by the 16S rRNA gene sequences, suggesting that this method is not appropriate to infer the phylogenetic relationships of *Leu*. *mesenteroides* strains. The phylogenetic analysis showed that *Leu*. *fallax* formed a clearly distinct phylogenetic lineage from the genera *Leuconostoc* as well as *Fructobacillus* (*F*.), suggesting that *Leu*. *fallax* may be reclassified as a new genus.

**Table 1:** General features of the genomes of *Leu*. *mesenteroides* strains and the type strains of closely related taxa used in this study^§^.

Strain name in GenBank (accession no.)	Genome status^a^ (No. of contigs)	Total size (Mb)	G + C content (%)	No. of genes	No. of pseudogenes	Isolation source	Completeness (%)^b^	Contamination (%)^b^
*Leu*. *mesenteroides* ssp. *jonggajibkimchii* DRC1506^T^ (CP014611–14)	C (4)	1.98	37.72	1,957	140	Kimchi	97.04	0.76
*Leu*. *mesenteroides* KFRI-MG (CP000574)	C (1)	1.90	37.70	1,884	23	Kimchi	96.78	0.40
*Leu*. *mesenteroides* P45 (JRGZ00000000)	D (6)	1.87	37.50	1,837	59	Pulque	92.93	2.07
*Leu*. *mesenteroides* Wikim17 (BBPK00000000)	D (41)	1.86	37.80	1,844	62	Kimchi	97.98	0.64
*Leu*. *mesenteroides* 406 (BCMP00000000)	D (69)	2.00	37.70	2,018	82	Airag	98.55	1.16
*Leu*. *mesenteroides* GL1 (LMXE00000000)	D (24)	1.82	38.10	1,717	41	Unknown	96.82	0.87
*Leu*. *mesenteroides* 213M0 (BCMO00000000)	D (58)	2.03	37.70	2,031	74	Airag	99.39	1.74
*Leu*. *mesenteroides* ssp. *mesenteroides* ATCC 8293^T^ (CP000414–15)	C (2)	2.08	37.66	2,061	30	Fermenting olives	99.50	0.11
*Leu*. *mesenteroides* ssp. *mesenteroides* J18 (CP003101–05)	C (5)	2.02	37.68	1,981	31	Kimchi	99.50	0.18
*Leu*. *mesenteroides* ssp. *mesenteroides* DRC0211 (CP013016–17, CP014602-4)	C (5)	2.02	37.80	2,082	55	Kimchi	99.50	1.15
*Leu*. *mesenteroides* ssp. *mesenteroides* BD3749 (CP014610)	C (1)	1.99	37.80	1,982	24	Unknown	98.60	2.07
*Leu*. *mesenteroides* ssp. *mesenteroides* LbE16 (LAYU00000000)	D (85)	2.04	37.50	2,097	123	Italian soft cheese	97.91	6.79
*Leu*. *mesenteroides* ssp. *dextranicum* DSM 20484^T^ (CP012009–10)	C (2)	1.85	38.04	1,876	95	Cheese	96.67	3.10
*Leu*. *mesenteroides* ssp. *dextranicum* LbE15 (LAYN00000000)	D (63)	2.01	37.60	2,045	103	Italian soft cheese	97.69	4.26
*Leu*. *mesenteroides* ssp. *cremoris* ATCC 19254^T^ (ACKV01000000)	D (126)	1.74	38.50	1,702	178	Hansen’s dried cheese	96.37	0.11
*Leu*. *mesenteroides* ssp. *cremoris* TIFN8 (ATAZ00000000)	D (173)	1.71	38.20	1,844	513	Dairy starter cultures	91.00	0.78
*Leu*. *mesenteroides* ssp. *cremoris* LbT16 (LAYV00000000)	D (65)	1.91	37.80	1,937	183	Italian soft cheese	97.48	2.19
*Leu*. *mesenteroides* ssp. *cremoris* T26 (JAUJ00000000)	D (130)	1.83	38.40	1,930	242	Undefined cheese	94.77	4.48
*Leu*. *suionicum* DSM 20241^T^ (CP015247–48)	C (2)	2.05	37.60	2,034	55	Unknown	97.44	2.57
*Leu*. *pseudomesenteroides* KCTC 3652^T^ (AEOQ00000000)	D (1160)	3.24	38.30	3,785	1,245	Cane juice		

^§^The genome analysis was carried out using the NCBI prokaryotic genome annotation pipeline (http://www.ncbi.nlm.nih.gov/genome/annotation_prok/).

^a^Genome status: D, draft genome sequence; C, complete genome sequence.

^b^Determined by CheckM.

### Relatedness based on average nucleotide identity (ANI) and *in silico* DNA-DNA hybridization (DDH) analyses and general features of *Leu*. *mesenteroides* genomes

Because the ANI cut-off value corresponding to 70% DDH threshold used as the gold standard for the delineation of prokaryotic species has been suggested to be approximately 95–96%^30–32^, ANI values among the genomes of *Leu*. *mesenteroides* strains, *Leu*. *suionicum*, and *Leu*. *pseudomesenteroides* were pair-wise calculated (Supplementary Fig. S1). The ANI analysis clearly showed that *Leu*. *pseudomesenteroides* KCTC 3652^T^ and *Leu*. *suionicum* DSM 20241^T^ shared less than the ANI cut-off value for the prokaryotic species delineation with other *Leu*. *mesenteroides* strains, corroborating the results of a previous study^2^. All *Leu*. *mesenteroides* strains shared higher ANI values (97.2–99.5%) than the ANI cut-off value, indicating that they belong to the same species. *In silico* DDH analysis also showed that all *Leu*. *mesenteroides* strains shared higher *in silico* DDH values (77.5–99.1%) than the 70% DDH threshold, whereas *Leu*. *suionicum* DSM 20241^T^ and *Leu*. *pseudomesenteroides* KCTC 3652^T^ shared clearly lower *in silico* DDH values than the 70% DDH threshold for the prokaryotic species delineation with other *Leu*. *mesenteroides* strains (Supplementary Fig. S2), confirming that strains DSM 20241^T^ and KCTC 3652^T^ represent new species distinct from other *Leu*. *mesenteroides* strains^2^.

The genome quality assessment conducted by the CheckM software (Table 1) showed that all genomes had ≥91.0% completeness and ≤6.8% contamination values, which satisfied the criteria for the genomes to be considered near complete (≥90%) with medium contamination (≤10%)^33^. However, the genome of *Leu*. *mesenteroides* ssp. *cremoris* TIFN8 contained numerous pseudogenes with frame shifts, most likely owing to incomplete genes by numerous contigs or high rates of sequencing error, and was therefore excluded from the next pan-genome analysis of *Leu*. *mesenteroides* strains. The average size and total gene number of *Leu*. *mesenteroides* genomes used for the pan-genome analysis were 1.94 ± 0.1 Mb and 1,940 ± 118, respectively. The genome of *Leu*. *mesenteroides* ssp. *cremoris* ATCC 19254^T^ was the smallest (1.74 Mb), whereas the genome of *Leu*. *mesenteroides* subsp. *mesenteroides* ATCC 8293^T^ was the largest (2.08 Mb). The G + C contents of *Leu*. *mesenteroides* strains ranged from 37.5% to 38.5%. The number of rRNA and tRNA genes in the completed genomes of *Leu*. *mesenteroides* strains were 12 and 68–70, respectively.

### Pan- and core-genome analysis of *Leu*. *mesenteroides*

**Figure 2:**
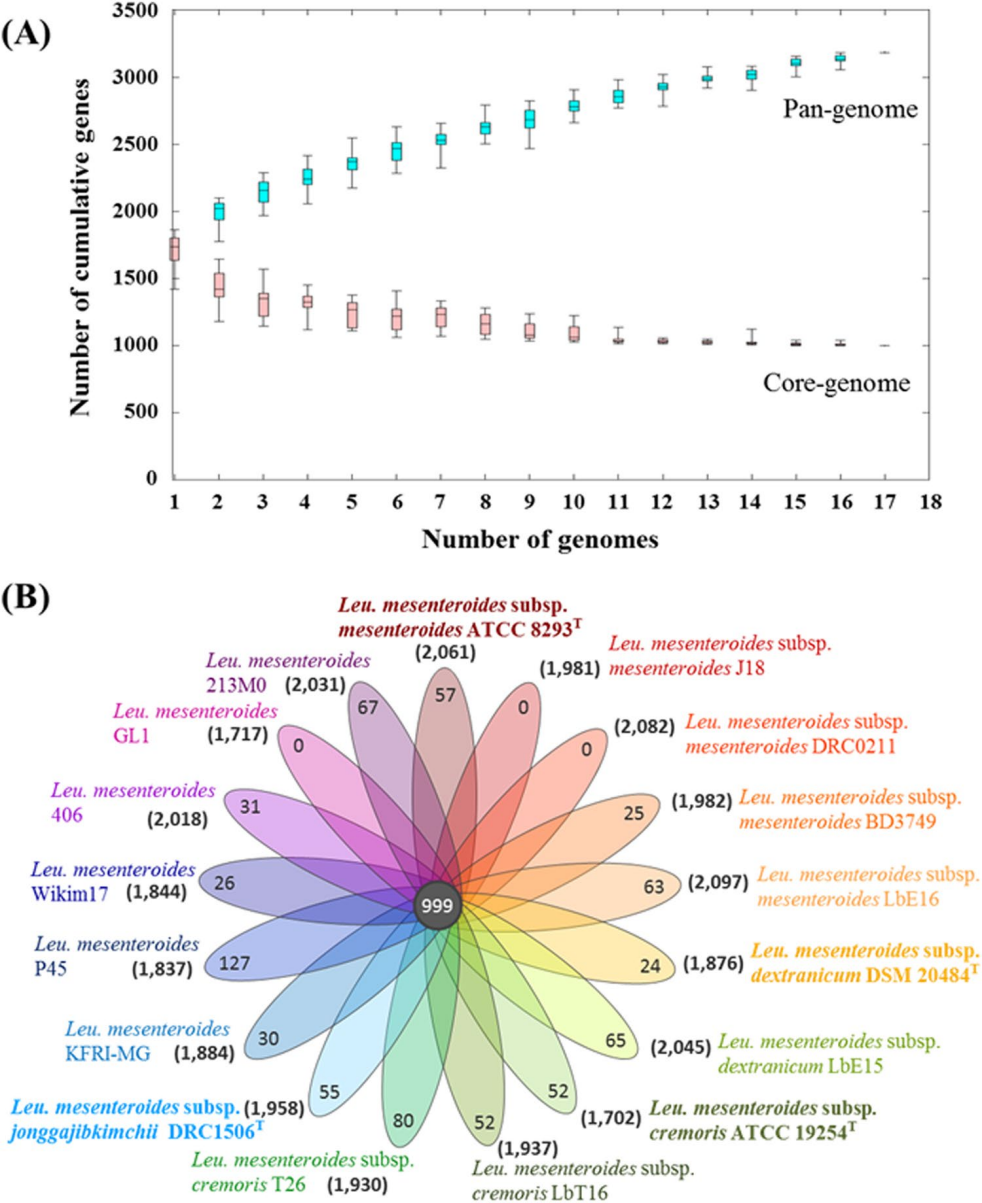
Pan- and core-genome plot (**A**) and flower plot diagram (**B**) of 17 *Leu*. *mesenteroides* strains. An ordered list of the 17 strains was randomly generated and 20 sets of the randomly ordered strains were subjected to pan- and core-genome analysis. The average number of core- and pan-genome sizes were plotted with standard deviations. The pan-genome represents the total genes of genomes in a subset sampled and the core-genome represents the genes shared by all genomes in the same subset. The flower plot diagram represents gene numbers in the core-genome (in the center) and unique-genome (in the petals) of *Leu*. *mesenteroides* pan-genome, and in the genome of each *Leu*. *mesenteroides* strain (in the parentheses). The type strains of *Leu*. *mesenteroides* subspecies are highlighted in bold.

The pan-genome is a powerful concept that can be used to effectively represent the genomic features of a bacterial lineage, and its analysis can provide insights into the genome dynamics and evolution of the lineage as well. Therefore, a pan-genome analysis for *Leu*. *mesenteroides* was performed using 17 *Leu*. *mesenteroides* genomes (Fig. 2A; Supplementary Fig. S3). The expected gene number for a given number of genomes in a pan-genome analysis can be estimated by a curve fitting represented by the Heaps’ law (n = k*N^-α^, where n is the expected gene number for a given number of genomes (N) and k is a constant to fit the specific curve)^34^. According to the Heaps’ law, an α < 1 is representative of an open pan-genome, meaning that each added genome contributes new genes and increases the pan-genome, whereas an α > 1 represents a closed pan-genome, in which the addition of new genomes does not significantly increase the pan-genome. The formula showed that the pan-genome of *Leu*. *mesenteroides* strains increases with an α of 0.23, indicating an open pan-genome and suggesting high evolutionary changes in *Leu*. *mesenteroides* genomes, through gene loss and gain or horizontal gene transfer (HGT) to adapt efficiently to new environmental conditions. The pan-genome for the 17 *Leu*. *mesenteroides* strains contained a total of 3,185 genes consisting of 999 genes in the core-genome, 1,432 genes in the accessory-genome (present in more than two strains), and 754 genes in the unique genome (Supplementary Table S1). Unique genes that differ among *Leu*. *mesenteroides* strains, which may reflect different niches and needs for the survival of *Leu*. *mesenteroides* strains and may be used in differentiating *Leu*. *mesenteroides* strains^35^.

Strain P45 was shown to have the highest number of unique genes (Fig. 2B; Supplementary Table 1), suggesting that this strain may have exchanged genes most actively with other bacterial groups. For example, strain P45 harbors a fructose-bisphosphate aldolase unique gene that is very closely related to the gene homolog of the *Leu*. *pseudomesenteroides* genome, with a 99% amino acid sequence identity, which indicates that strain P45 might have gained the gene by HGT from *Leu*. *pseudomesenteroides*. Strain P45 harbors another unique gene encoding a peptide ABC transporter ATP-binding protein, possibly conferring it an antibacterial activity as bacteriocin^36^. The gene has the highest amino acid sequence identity (83%) with a gene homolog of *F. pseudoficulneus*, indicating that the gene might have also been transferred by HGT. Strain LbT16 has four lactate dehydrogenase genes (ldh), including a unique gene (Supplementary Table 1). This unique ldh gene is most closely related to an ldh gene homolog found on the genome of *Leu*. *pseudomesenteroides*, with a 98% amino acid sequence identity, which implies that strain LbT16 might have acquired the gene by HGT. These results suggest that HGT may be one of major mechanisms to foster genome variations or speciation in *Leu*. *mesenteroides* strains and their relatives^37^.

### Relatedness of *Leu*. *mesenteroides* strains based on core-genomes and molecular phenotypes

**Figure 3:**
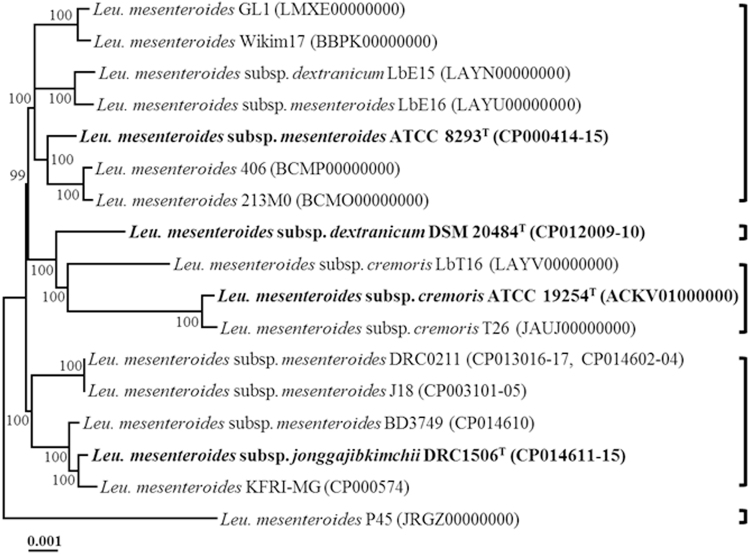
A phylogenetic tree with bootstrap values (1,000 replicates) reconstructed using the concatenated amino acid sequences of *Leu*. *mesenteroides* core-genome (999 genes) showing the relationships among *Leu*. *mesenteroides* strains. Strain names as described in GenBank or validated names are used in the tree and the type strains are highlighted in bold. The bar indicates 0.001 substitutions per site.

To infer the phylogenetic relationships among *Leu. mesenteroides* strains, a phylogenetic tree was constructed using the concatenated amino acid sequences of 999 genes in the core-genome (Fig. 3). Unlike the phylogenetic tree based on 16S rRNA gene sequences, the phylogenetic tree based on the core-genome showed that *Leu*. *mesenteroides* strains have been more clearly separated into different phylogenetic lineages, which were relatively consistent with the hierarchical clustering based on ANI and *in silico* DDH values of Figs 2 and 3. All strains of the phylogenetic lineage containing *Leu*. *mesenteroides* subsp. *jonggajibkimchii* DRC1506^T^ as the type subspecies were isolated from fermented vegetables (mainly kimchi), which suggests that these lineage members became well adapted to the fermentation environments of vegetables. Strain P45, isolated from a traditional Mexican alcoholic fermented beverage and having the highest number of unique genes, formed a phylogenetic lineage clearly distinct from other *Leu*. *mesenteroides* strains, which may reflect a different habitat of strain P45 (alcoholic fermented beverage) from those of other *Leu*. *mesenteroides* strains (fermented vegetables).

**Figure 4:**
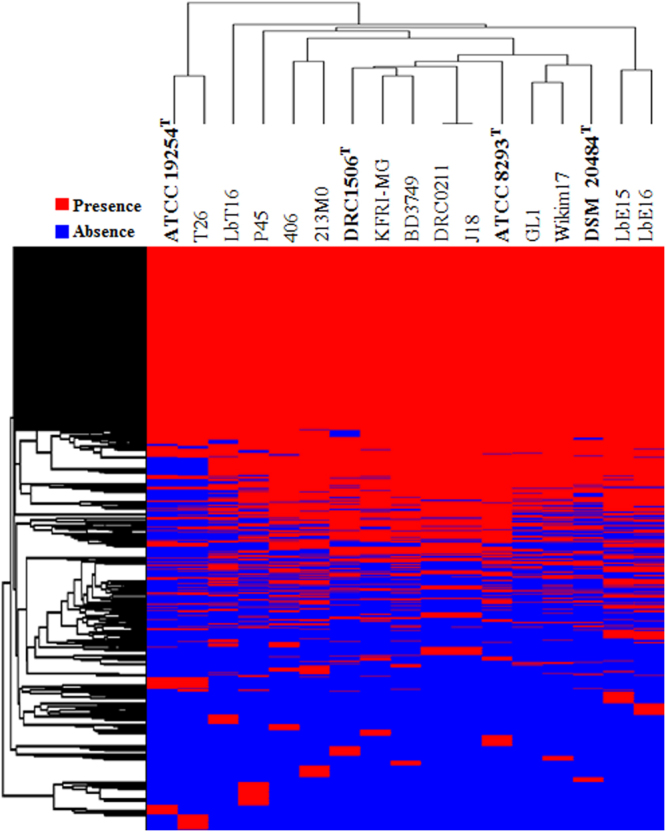
Heat-map and hierarchical clustering of 17 *Leu*. *mesenteroides* strains based on the presence (red) or absence (blue) of genes. The type strains of *Leu*. *mesenteroides* subspecies are highlighted in bold.

Gene gain or loss, and HGT between the genomes of organisms occur continuously during the evolutionary processes. It is generally accepted that closely related organisms share more orthologous genes, suggesting that evolutionary relationships among *Leu*. *mesenteroides* can be inferred by the presence/absence of orthologous genes. A total of 3,185 genes were identified from the genomes of 17 *Leu*. *mesenteroides* strains. However, the heat map based on the presence/absence of these genes showed that the hierarchical clustering was slightly different from those based on ANI and *in silico* DDH values, and core-genomes (Fig. 4), indicating that gene gain or loss and HGT may have occurred among *Leu*. *mesenteroides* strains as well as other clade organisms.

### Clusters of orthologous groups (COG) analysis of *Leu*. *mesenteroides* genomes

**Figure 5:**
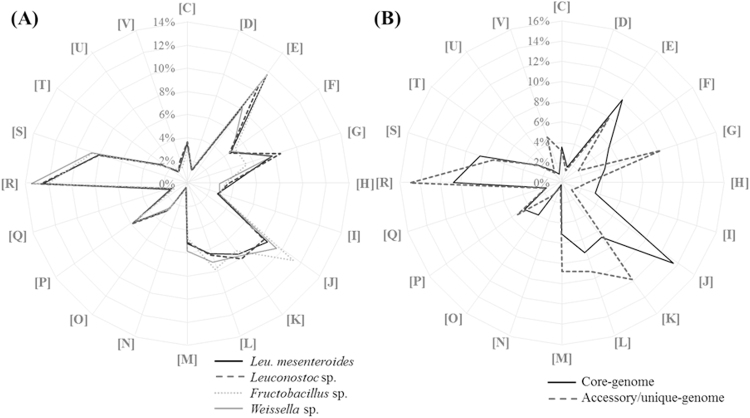
Comparison of COG functional categories in the pan-genomes of *Leu*. *mesenteroides* strains and closely related bacterial taxa (*Leuconostoc* species except for *Leu*. *mesenteroides, Fructobacillus* species, and *Weissella* species) (**A**) and distribution of the COG functional categories in the core- and accessory/unique-genome of *Leu*. *mesenteroides* strains (**B**). The alphabetic codes represent COG functional categories as follows: C, energy production and conversion; D, cell division and chromosome partitioning; E, amino acid transport and metabolism; F, nucleotide transport and metabolism; G, carbohydrate transport and metabolism; H, coenzyme metabolism; I, lipid metabolism; J, translation, ribosomal structure, and biogenesis; K, transcription; L, DNA replication, recombination, and repair; M, cell envelope biogenesis, outer membrane; N, cell motility and secretion; O, post-translational modification, protein turnover, and chaperones; P, inorganic ion transport and metabolism; Q, secondary metabolite biosynthesis, transport, and catabolism; R, general function prediction only; S, function unknown; T, signal transduction mechanisms; U, intracellular trafficking, secretion, and vesicular transport; V, defense mechanisms.

The analysis of functional categories enriched in a pan-genome may provide valuable clues in identifying the selective pressures and evolutionary developments of a bacterial lineage^22, 38^. Therefore, all genes in the genome of *Leu*. *mesenteroides* strains were functionally classified based on their COG categories, and their average abundances were compared with those in the genomes of closely related taxa (*Leuconostoc* species except for *Leu*. *mesenteroides*, *Fructobacillus* species, and *Weissella* species) (Fig. 5A). Functional genes belonging to COG categories involved in carbohydrate and energy metabolism, including amino acid transport and metabolism (E); carbohydrate transport and metabolism (G); translation, ribosomal structure, and biogenesis (J); transcription (K); and general function prediction only (R) were enriched in the genomes of *Leu*. *mesenteroides* strains (>6%). The distribution of functional genes into COG categories in *Leu*. *mesenteroides* was relatively similar to those of closely related LAB, suggesting that the distribution of the COG categories may be a general feature of the genomes of LAB that have adapted to similar environmental conditions.

A functional characterization of the core- and accessory/unique genomes of *Leu*. *mesenteroides* strains was also performed, by assigning the core- and accessory/unique genome to a COG functional category, and some clear differences between the core- and accessory/unique-genome were observed (Fig. 5B). As expected, genes involved in housekeeping processes including translation, ribosomal structure, and biogenesis (J); amino acid transport and metabolism (E); nucleotide transport and metabolism (F); lipid transport and metabolism (I); and posttranslational modification, protein turnover, and chaperones (O) were more enriched in the core-genome than in the accessory/unique genome. In contrast, COG categories related to energy metabolism or DNA repair, including carbohydrate transport and metabolism (G); transcription (K); DNA replication, recombination, and repair (L); cell wall/membrane/envelope biogenesis (M); general function prediction only (R); and defense mechanisms(V) were more abundant in the accessory/unique-genome than in the core-genome. Higher abundance of genes corresponding to carbohydrate transport and metabolism (G) in the accessory/unique-genome than in the core-genome suggests that the fermentation features of *Leu*. *mesenteroides* strains for carbohydrate compounds differ among *Leu*. *mesenteroides* strains.

Most *Leuconostoc* species are intrinsically resistant to vancomycin due to a peculiar cell wall structure with d-lactate instead of d-alanine at the terminal end of peptidoglycans, and not by general antibiotic resistance mechanisms^18, 39, 40^. It was reported that the d-alanyl-d-alanine ligase that synthesizes d-alanyl-d-alanine in *Leu*. *mesenteroides* can also synthesize d-alanyl-d-lactate of the peptidoglycan^41, 42^. Our analysis revealed that a gene encoding d-alanyl-d-alanine ligase (Enzyme Commission (EC) 6.3.2.4) was identified from the genomes of all *Leu*. *mesenteroides* strains at the core genome, which confirms that the vancomycin resistance is a common species feature of *Leu*. *mesenteroides*. Because there were some clinical reports of the possible pathogenicity of *Leu*. *mesenteroides*, we investigated the presence of virulence genes from the pan-genome of *Leu*. *mesenteroides* strains. The genes encoding hemolysin and hemolysin III that have been considered as potential virulence genes were identified from the core genome of *Leu*. *mesenteroides* strains^43, 44^ — any other known potential virulence genes were not identified from the pan-genome of *Leu*. *mesenteroides* strains. The two hemolysin and hemolysin III-coding genes were also identified from the genomes of LAB such as *L. rhamnosus* GG, *L. plantarum*, and *L. sakei* that are well-known as safe probiotics. However, our tests showed that *Leu*. *mesenteroides* strains J18, DRC0211, DRC1506^T^, and ATCC 8293^T^ did not show any hemolytic activity (data not shown), which may suggest that *Leu*. *mesenteroides* strains do not have pathogenic activities related to hemolysin and hemolysin III genes. However, further studies are needed to investigate the pathogenic possibility of *Leu*. *mesenteroides* strains as an infectious agent in humans.

### Kyoto encyclopedia of genes and genomes (KEGG) and fermentative metabolic pathways of *Leu*. *mesenteroides*

**Figure 6:**
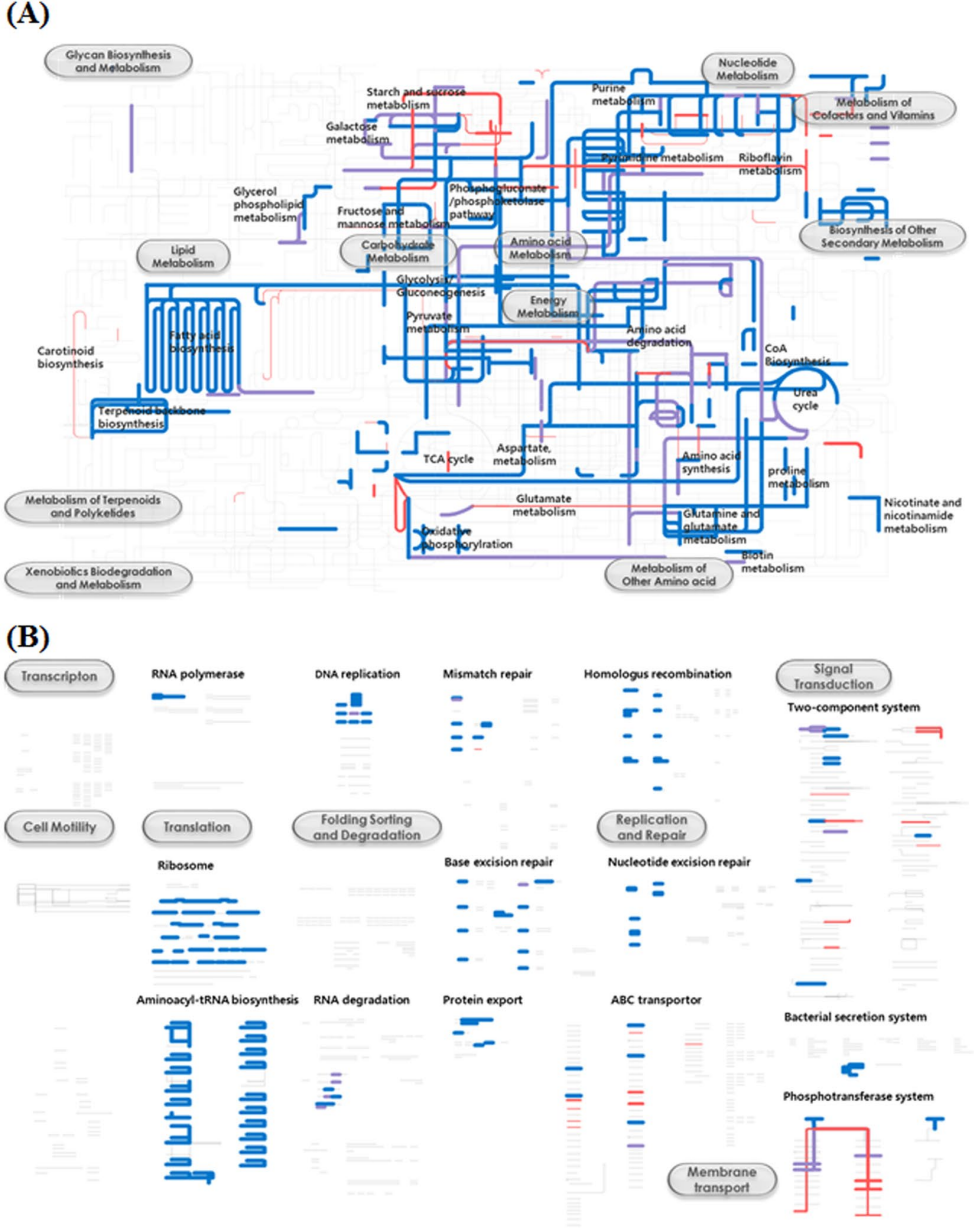
Metabolic (**A**) and regulatory (**B**) pathways of *Leu*. *mesenteroides* strains. The pathways were generated using the iPath v2 module based on KEGG Orthology numbers of genes identified from the genomes of 17 *Leu*. *mesenteroides* strains. Metabolic pathways identified from all 17 genomes, belonging to the core-genome, are depicted in blue and metabolic pathways identified from 15–16 genomes, belonging to the soft core-genome, are depicted in violet. Metabolic pathways identified from 1–14 genomes, belonging to accessory/unique-genome, are depicted in red. Line thickness is proportional to the numbers of *Leu*. *mesenteroides* strains harboring the metabolic pathways.

To investigate the metabolic features and diversities of *Leu*. *mesenteroides*, all functional genes of 17 *Leu*. *mesenteroides* strains were cumulatively mapped onto KEGG pathways (Fig. 6). Some sequencing errors in the genome sequencing are generated by base over- or under-call, resulting in frame shift errors in gene sequences, which eventually cause the incorrect description of normal genes as pseudogenes during the genome annotation process. It is inferred that some portions of pseudogenes shown in Table 1 might be caused by genome sequencing errors. Therefore, in this study, genes identified from 15–16 *Leu*. *mesenteroides* genomes were defined as soft core-genome and the metabolic pathways identified from more than 15 genomes were considered as common metabolisms in *Leu*. *mesenteroides*.

The KEGG pathway analysis showed that all *Leu*. *mesenteroides* strains harbors the 6-phosphogluconate/phosphoketolase pathway, pyruvate metabolism, and incomplete glycolysis pathway and TCA cycle (Fig. 6A), representing the obligate heterolactic fermentation of *Leu*. *mesenteroides*. In addition, the KEGG analysis showed that *Leu*. *mesenteroides* strains have fatty acid biosynthesis, galactose degradation, fructose and mannose metabolism, purine and pyrimidine metabolism, amino acid metabolism, coenzyme A biosynthesis, and oxidative phosphorylation as common metabolic pathways. Genes associated with starch and sucrose metabolism (Fig. 6A), phosphotransferase systems (PTS), two-component systems, and ABC transporter (Fig. 6B) are present in the accessory- or unique-genome, which suggests that carbohydrate metabolisms may be different between *Leu*. *mesenteroides* strains. The KEGG analysis also showed that riboflavin biosynthesis may be different depending on *Leu*. *mesenteroides* strains.

**Figure 7:**
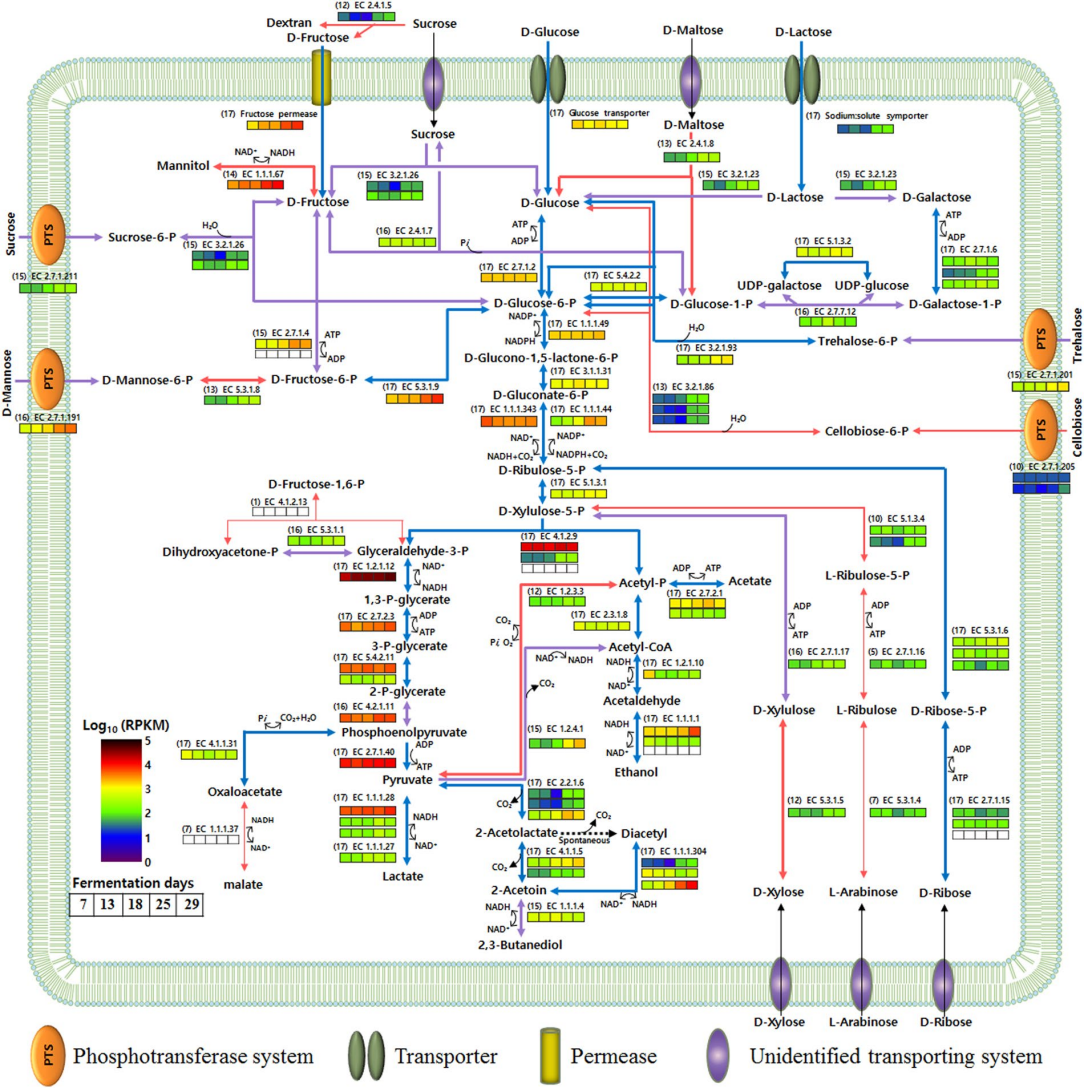
Proposed fermentative metabolic pathways of *Leu*. *mesenteroides* for carbohydrates and their transcriptional expressions during kimchi fermentation. Metabolic pathways that were present in all *Leu*. *mesenteroides* strains are depicted in blue (core-genome) and metabolic pathways that were present in 15–16 *Leu*. *mesenteroides* strains are depicted in violet (soft core-genome). Metabolic pathways that were present in 1–14 *Leu*. *mesenteroides* strains are depicted in red (unique- or accessory-genome). Line thickness in the pathways is proportional to the number of *Leu*. *mesenteroides* strains harboring the corresponding genes, which are indicated in parentheses before EC numbers. Carbohydrate transport systems with black arrows indicate unidentified transporting systems that may be present in *Leu*. *mesenteroides* genomes. The transcriptional expressions were visualized by heatmaps based on their RPKM values and the white boxes represent no transcriptional expression during kimchi fermentation. Kimchi samples for the metatranscriptomic analysis were obtained at 7, 13, 18, 25, and 29 days. UDP, uridine diphosphate.

Based on the predicted KEGG pathways and BLASTP analysis of genes related to carbohydrate fermentation, the fermentative metabolic pathways of *Leu*. *mesenteroides* strains for carbohydrates were reconstructed (Fig. 7) and genomes deficient for genes encoding proteins forming the reconstructed fermentative metabolic pathways are listed in supplementary Table S2. Diverse carbohydrate transport systems, including sugar phosphotransferase systems (PTS), transporters, and permeases were found from the genomes of *Leu*. *mesenteroides* strains, indicating that *Leu*. *mesenteroides* can metabolize diverse carbohydrates. Genes associated with the metabolism of glucose, fructose, ribose, lactose, sucrose, mannose, and trehalose were identified from the core- or soft core-genome of *Leu*. *mesenteroides*, indicating that they may be common carbohydrates metabolized fermentatively by *Leu*. *mesenteroides*. Conversely, genes associated with the metabolism of maltose, xylose, arabinose, and cellobiose were identified from the accessory genome of *Leu*. *mesenteroides*, indicating that the metabolic ability of *Leu*. *mesenteroides* for them may differ between *Leu. mesenteroides* strains. Most *Leu*. *mesenteroides* strains have been reported to produce dextran, a viscous glucose homopolysaccharide with predominantly α-(1,6)-glycosidic linkages, and this can be a common feature of *Leu*. *mesenteroides* ^45, 46^. However, a gene encoding dextransucrase (a glycosyltransferase, EC 2.4.1.5) that synthesizes dextran from sucrose was found from the genomes of 11 *Leu*. *mesenteroides* strains, which suggests that the dextran production differs between *Leu*. *mesenteroides* strains and is not a common feature of *Leu*. *mesenteroides*.

The carbohydrate metabolic capabilities of *Leu*. *mesenteroides* strains shown in the reconstructed fermentative metabolic pathways were verified using the type strains of four *Leu*. *mesenteroides* subspecies (ssp. *mesenteroides*, ssp. *jonggajibkimchii*, ssp. *dextranicum*, and ssp. *cremoris*) harboring different carbohydrate transport systems and metabolic genes (Supplementary Table S3). All test strains had capabilities to ferment d-glucose, sucrose, and gluconate. The types strains of *Leu*. *mesenteroides* subsp. *mesenteroides* and *Leu*. *mesenteroides* ssp. *jonggajibkimchii* that harbor diverse carbohydrate transport systems had capabilities to ferment various carbohydrates including trehalose, d-maltose, d-mannose, d-lactose, l-arabinose, d-xylose, mannitol, and d-ribose, while the type strain of *Leu*. *mesenteroides* subsp. *cremoris* deficient in genes encoding fructokinase (EC 2.7.1.4), *β*-galactosidase (EC 3.2.1.23), PTS cellobiose transporter subunit IIABC (EC 2.7.1.205), 6-phospho-*β*-glucosidase (3.2.1.86), maltose phosphorylase (EC 2.4.1.8), and mannose-6-phosphate isomerase (EC 5.3.1.8) did not have a capability to ferment various carbohydrates including fructose, d-lactose, cellulobios, maltose, d-mannose, and mannitol. In addition, the type strain of *Leu*. *mesenteroides* subsp. *cremoris* deficient in glycosyltransferase (EC 2.4.1.5) gene did not produce dextran, while other three *Leu*. *mesenteroides* subspecies harboring the gene produced dextran. These test results were in good accordance with their genome-based metabolic properties shown in Fig. 7. However, some fermentation capabilities of the test strains were different from their genome-based metabolic properties. For example, the type strains of *Leu*. *mesenteroides* ssp. *jonggajibkimchii* and *Leu*. *mesenteroides* subsp. *dextranicum* deficient in genes encoding l-arabinose isomerase (EC 5.3.1.4), ribulokinase (EC 2.7.1.16), and ribulose phosphate epimerase (EC 5.1.3.4) had an ability to ferment l-arabinose. In addition, the type strain of *Leu*. *mesenteroides* ssp. *jonggajibkimchii* did not ferment cellobiose although the strain harbors a cellobiose PTS system in its genome, which suggests that we need further studies to explore the functions and expressions of genes and their metabolic properties in *Leu*. *mesenteroides* strains.

All genes involved in the 6-phosphogluconate/phosphoketolase pathway, representing the heterolactic fermentation to produce lactate, ethanol, and carbon dioxide were retrieved from the core-genome of *Leu*. *mesenteroides* strains (Fig. 7), confirming that the heterolactic fermentation is a common metabolic feature of *Leu*. *mesenteroides*. Fructose-bisphosphate aldolase (EC 4.1.2.13), which splits fructose 1,6-bisphosphate into dihydroxyacetone-phosphate and glyceraldehyde-3-phosphate, is known as a key enzyme of homofermentative LAB, but deficient in heterofermentative LAB. Despite this, a gene encoding fructose-bisphosphate aldolase was found from *Leu*. *mesenteroides* strain P45 harboring the complete heterofermentative pathway. However, because a gene encoding 6-phosphofructokinase (EC 2.7.1.11), another key enzyme for homolactic fermentation that catalyzes the phosphorylation of fructose-6-phosphate to fructose 1,6-bisphosphate, is deficient in strain P45, *Leu*. *mesenteroides* strains most likely perform only heterolactic fermentation. It is inferred that the fructose-bisphosphate aldolase coding gene in strain P45 might have been accidentally acquired from a homofermentative LAB through a lateral gene transfer. Pyruvate, originating from glyceraldehyde-3-phosphate, a product of d-xylulose-5-phosphate cleavage by phosphoketolase (EC 4.1.2.9) in heterolactic fermentation, is converted into lactate by lactate dehydrogenases with the regeneration of NAD^+^. Figure 7 shows that *Leu*. *mesenteroides* strains harbor three copies of d-lactate dehydrogenase (EC 1.1.1.28) and one copy of l-lactate dehydrogenase (EC 1.1.1.27) in the core-genome, which may support previous reports that *Leu*. *mesenteroides* strains produce more d-lactate than l-lactate^47^. Our genome analysis revealed that a gene encoding the membrane-bound d-lactate dehydrogenase (EC. 1.1.5.12) that is distinct from other general d- or l-lactate dehydrogenases was additionally identified from two *Leu*. *mesenteroides* strains (DSM 20284^T^ and Wikim17). It was reported that this membrane-bound d-lactate dehydrogenase oxidizes d-lactate to pyruvate with the generation of two electrons that are eventually transferred into oxygen through an electron transport system under aerobic conditions, not anaerobic conditions^48, 49^, which suggests that the enzyme might not be associated with the metabolite production during fermentation.

The reconstructed fermentative metabolic pathways showed that, in addition to the conversion of pyruvate to lactate, pyruvate can be alternatively converted into other fermentative metabolites in *Leu*. *mesenteroides* strains. Diacetyl and acetoin are known to be important cheese flavors in dairy products^50^. Genes encoding acetolactate synthase (EC 2.2.1.6), acetolactate decarboxylase (EC 4.1.1.5), and diacetyl reductase (EC 1.1.1.304) that convert pyruvate to diacetyl and/or acetoin were found from the core-genome of *Leu*. *mesenteroides* strains, indicating that diacetyl or acetoin may be also common major flavoring compounds in fermented foods where *Leu*. *mesenteroides* strains are involved. However, 2,3-butanediol dehydrogenase (EC 1.1.1.4) that converts 2-acetoin to 2,3-butanediol with the regeneration of NAD^+^ was also found from the soft-core genome. This may suggest that this enzyme can somewhat weaken cheese flavoring intensities in foods fermented by *Leu*. *mesenteroides* strains; fermented foods such as kimchi and sauerkraut fermented by *Leu*. *mesenteroides* strains do not smell strongly of cheese flavoring. The reconstructed fermentative metabolic pathways also showed that pyruvate can be converted into acetyl-CoA with the production of carbon dioxide and NADH by the pyruvate dehydrogenase complex (EC 1.2.4.1). A gene encoding the pyruvate dehydrogenase complex was found from the soft core-genome, indicating that acetyl-CoA production from pyruvate may be a common metabolic pathway in *Leu*. *mesenteroides* strains. Finally, a gene encoding pyruvate oxidase (EC 1.2.3.3) was found from 12 *Leu*. *mesenteroides* genomes, enabling the strains to convert pyruvate into acetyl phosphate (acetyl-P) and carbon dioxide when oxygen is available^51, 52^.

Acetyl-P is another split product of d-xylulose-5-phosphate by phosphoketolase in the heterolactic fermentation, and it is eventually converted into ethanol with the regeneration of NAD^+^ or acetate with the production of ATP as the final products, indicating that the final products are decided by reduction potentials (NADH concentrations) inside the cell. Heterofermentative LAB that produce lactic acid, ethanol, and carbon dioxide from glucose generate one mole of ATP per mole of glucose, meaning that they are less competitive than homofermentative LAB that produce 2 moles of ATP per mole of glucose. The reconstructed fermentative metabolic pathways showed that 14 *Leu*. *mesenteroides* genomes harbor a gene encoding mannitol dehydrogenase (EC 1.1.1.67) that produces mannitol^9^. The remaining three genomes (strains ATCC 19254^T^, DSM 20484^T^, and T26) also harbor the gene as a pseudogene, possibly having mannitol dehydrogenase activity because the annotations as pseudogenes can be caused by sequencing errors. Carvalheiro *et al*.^53^ reported that strains ATCC 19254^T^ and DSM 20484^T^ produced mannitol through the consumption of fructose, suggesting that they have a mannitol dehydrogenase activity. These results suggest that mannitol production may be a common species feature of *Leu*. *mesenteroides*. Mannitol is produced through fructose reduction with the consumption of NADH, which may cause the production of acetate instead of ethanol due to the lack of NADH; one mole of ATP will be additionally produced per 2 moles of mannitol production. This suggests that heterofermentative *Leu*. *mesenteroides* with mannitol dehydrogenase activity can be as competitive as homofermentative LAB in terms of energy production during fermentation of vegetables containing fructose^8^. This is in accordance with the previous results that *Leu*. *mesenteroides* was dominant in vegetable fermentations such as kimchi and sauerkraut, which contain fructose^7, 54, 55^.

### Metabolic properties of *Leu*. *mesenteroides* during kimchi fermentation

**Figure 8:**
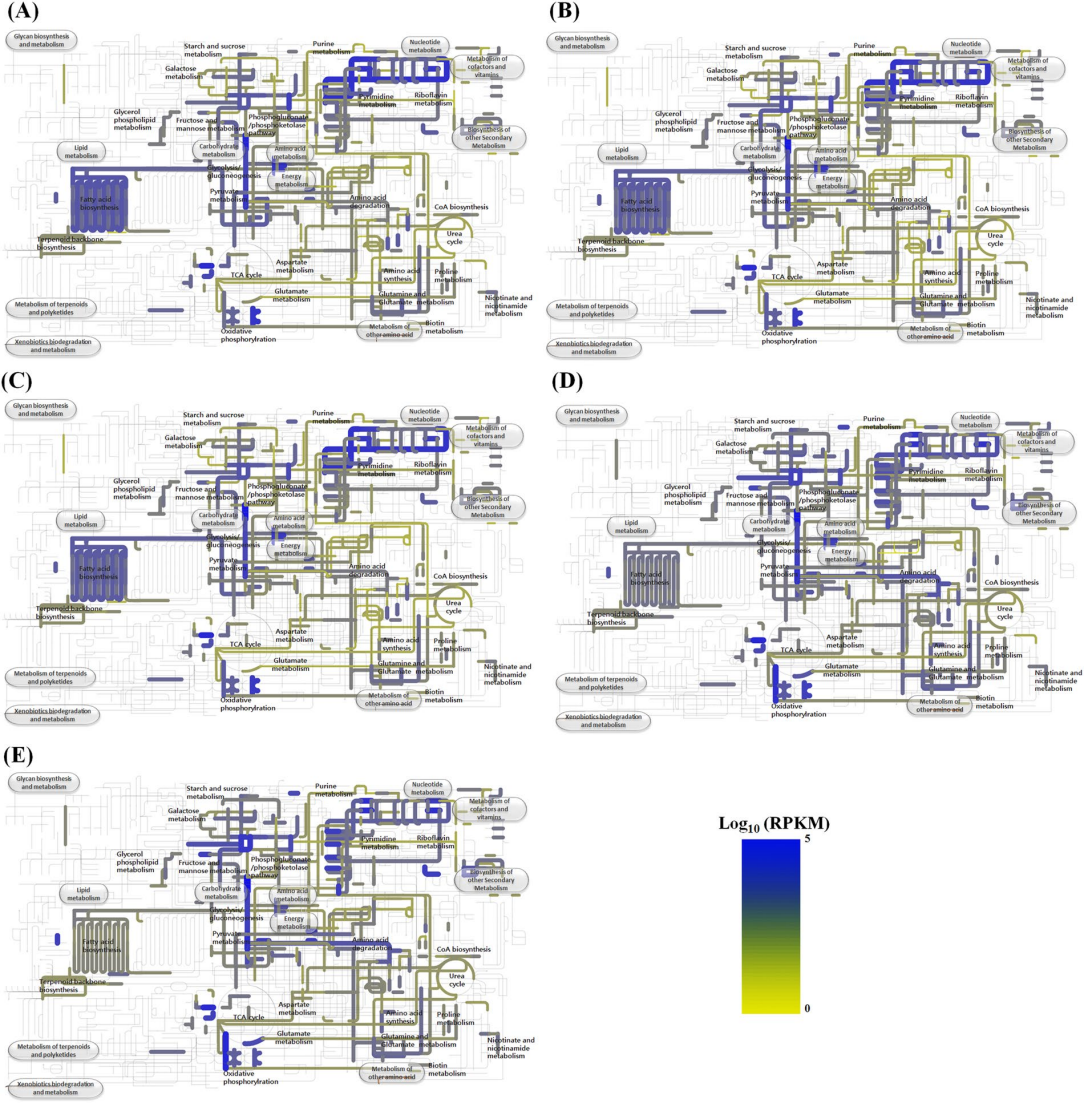
Transcriptional expressions of the metabolic pathways of *Leu*. *mesenteroides* at 7 (**A**), 13 (**B**), 18 (**C**), 25 (**D**), and 29 (**E**) days during kimchi fermentation. The metabolic pathways were generated using the iPath v2 module based on KEGG Orthology numbers identified from the pan-genome of *Leu*. *mesenteroides* strains. The transcriptional expression levels of the metabolic pathways are depicted by line thickness and color change based on their RPKM values (based on a log_2_ scale).

Traditional kimchi that is fermented naturally at low temperatures without any starter is a complex system, with dynamic biological and biochemical changes during fermentation. Because kimchi fermentation is accomplished by a succession of naturally occurring different LAB, fermentative metabolic features of microbial communities during kimchi fermentation process are different every time, which makes it difficult to consistently produce standardized kimchi with high quality. Until now, rational and systematic approaches to control kimchi fermentation for the production of kimchi with uniform quality have not been developed because the understanding of kimchi microbial communities during fermentation has not yet been accomplished. Therefore, comprehensive investigation on the fermentative metabolic features of kimchi LAB during fermentation is indispensable to control kimchi fermentation^7, 8, 12^. With metatranscriptomic analysis, it is relatively easy to investigate the metabolic features of microbial communities in fermented foods such as kimchi, because these communities are not so complex as those in other natural environments^56–59^. Therefore, in this study, a transcriptomic analysis was performed to examine the metabolic features of *Leu*. *mesenteroides* during kimchi fermentation. Relative abundances (%) of mRNA sequencing reads mapped to the genomes of *Leu*. *mesenteroides* strains for total LAB mRNA sequencing reads during the kimchi fermentation were calculated. The relative abundances were high at the early kimchi fermentation period and decreased to the lowest value at 18 days as the fermentation progressed (Supplementary Fig. S4), suggesting that members of *Leu*. *mesenteroides* are more responsible for kimchi fermentation during the early fermentation period. The metabolic properties of *Leu*. *mesenteroides* during kimchi fermentation were investigated by metabolic mapping of the *Leu*. *mesenteroides* mRNA sequencing reads onto the KEGG pathways of *Leu*. *mesenteroides* strains (Fig. 8). The transcriptomic analysis showed that genes associated with carbohydrate metabolisms, nucleotide metabolism, fatty acid biosynthesis, oxidative phosphorylation, riboflavin metabolism, and glutamine and glutamate metabolism were highly expressed during kimchi fermentation. Genes associated with fatty acid biosynthesis, nucleotide metabolism, and amino acid metabolism, probably more related to cell growth, were up-regulated in *Leu*. *mesenteroides* during the early kimchi fermentation period, which may explain why *Leu*. *mesenteroides* is more abundant at that stage. Conversely, genes associated with oxidative phosphorylation, biosynthesis of other secondary metabolisms, and glutamine and glutamate metabolism were up-regulated during the late kimchi fermentation period. Genes associated with the biosynthesis of riboflavin were highly expressed during the entre kimchi fermentation, suggesting that *Leu*. *mesenteroides* may be an important producer of riboflavin during kimchi fermentation.

The fermentative metabolic features of *Leu*. *mesenteroides* for carbohydrates were more thoroughly investigated by the transcriptomic analysis of respective genes corresponding to the fermentative metabolic pathways of *Leu*. *mesenteroides* during kimchi fermentation (Fig. 7). The transcriptomic analysis showed that *Leu*. *mesenteroides* in the kimchi samples performed heterolactic fermentation using diverse carbohydrates including glucose, fructose, mannose, trehalose, sucrose, maltose, and cellobiose. All genes corresponding to the heterolactic fermentation to produce lactate, ethanol, and carbon dioxide were highly expressed during the entire kimchi fermentation. The transcriptomic analysis showed that genes associated with the transport of glucose, fructose, and mannose were highly expressed, which suggests that glucose, fructose, and mannose may be major carbon sources during kimchi fermentation. A gene associated with a glucose transporter had relatively high expression during the early kimchi fermentation period, whereas genes associated with the transport of other carbon sources such as fructose, mannose, trehalose, and sucrose were highly expressed during the late fermentation period, which suggests that *Leu*. *mesenteroides* uses glucose more preferably than other carbon sources during kimchi fermentation, similar to other LAB^60^.

*Leu*. *mesenteroides* is known to be more responsible for kimchi fermentation during the early fermentation period, and its contribution to kimchi fermentation decreases gradually as the fermentation progresses^3, 8^. Metatranscriptomic analysis also showed that the transcriptional activity of *Leu*. *mesenteroides* was high in the early period and decreased gradually as kimchi fermentation progressed^7^ (supplementary Fig. S4). In addition, genes associated with cell growth such as fatty acid biosynthesis, nucleotide metabolism, and amino acid metabolism were up-regulated during the early kimchi fermentation period (Fig. 8). However, genes associated with fermentative metabolisms of *Leu*. *mesenteroides* for carbohydrates, probably more related to energy production, were generally up-regulated during the late fermentation period (Fig. 7), which may be due to the energy needs of *Leu*. *mesenteroides* under stress conditions of kimchi (e.g., low pH, depletion of carbon sources) towards the end of fermentation^61^. A gene encoding pyruvate oxidase that catalyzes the conversion between pyruvate and acetyl-P was expressed during the kimchi fermentation, although the expression levels were relatively low. Because the conversion from pyruvate to acetyl-P occurs when oxygen is available and kimchi fermentation is processed under anaerobic conditions, the conversion from acetyl-P to pyruvate may be the major direction of pyruvate oxidase in kimchi fermentation. The transcriptome analysis showed that the genes encoding fructose-bisphosphate aldolase (EC 4.1.2.13) and malate dehydrogenase (EC 1.1.1.37), identified from only one genome and seven genomes of *Leu*. *mesenteroides* strains, respectively, were not expressed during the entire kimchi fermentation period, suggesting that *Leu*. *mesenteroides* strains in kimchi may not harbor the genes. The transcriptome analysis also showed that genes encoding acetolactate synthase (EC 2.2.1.6), acetolactate decarboxylase (EC 4.1.1.5), and diacetyl reductase (EC 1.1.1.304) that convert pyruvate to diacetyl and/or acetoin were expressed during the kimchi fermentation, indicating that diacetyl and/or acetoin may be major flavoring compounds in kimchi. However, a gene encoding butanediol dehydrogenase that converts 2-acetoin to 2,3-butanediol was also highly expressed, which may explain the weak cheese flavoring intensities of fermented kimchi.

## Conclusions

In this study, we investigated the genomic diversity and features of *Leu*. *mesenteroides* strains performing heterolactic fermentation by using the pan-genome of *Leu*. *mesenteroides* strains and analyzing their metabolic features through the COG and KEGG analyses. In addition, we reconstructed the fermentative metabolic pathways of *Leu*. *mesenteroides* and examined its fermentative metabolic features for various carbohydrates through a metatranscriptomic analysis during kimchi fermentation. This study shows that the pan-genomic and metatranscriptomic analyses of kimchi LAB provide a better understanding of their comprehensive genomic and metabolic features during kimchi fermentation.

## Materials and Methods

### Genomes used in this study and phylogenetic analysis based on 16S rRNA gene sequences

At the time of writing (December 2016), the genome sequences of all *Leu*. *mesenteroides* strains and the type strains of *Leu*. *suionicum* and *Leu*. *pseudomesenteroides*, close relatives of *Leu*. *mesenteroides*, available in GenBank were downloaded and quality-assessed using the CheckM software (ver. 1.0.4)^33^. To infer evolutionary relationships among *Leu*. *mesenteroides* strains and their relative taxa, a phylogenetic analysis based on 16S rRNA gene sequences was conducted. The 16S rRNA gene sequences of all *Leu*. *mesenteroides* strains with whole genome sequencing information in GenBank and their closely related type strains were aligned using the Infernal secondary-structure aware aligner, available in the Ribosomal Database Project (http://rdp.cme.msu.edu/)^62^. A phylogenetic tree based on the 16S rRNA gene sequences was constructed using the neighbor-joining algorithm of the PHYLIP software (ver. 3.695)^63^. All genome sequences of *Leuconostoc* sp.*, Fructobacillus* sp., and *Weissella* sp. were also downloaded from the GenBank database to compare with the genomes of *Leu*. *mesenteroides*.

### ANI and *in silico* DDH analyses

Genome-based ANI and *in silico* DDH analyses were used to evaluate the relatedness among *Leu*. *mesenteroides* strains and the type strains of *Leu*. *suionicum* and *Leu*. *pseudomesenteroides*. The pair-wise ANI values among the genomes, including chromosomes and plasmids, of all *Leu*. *mesenteroides* strains and the type strains of *Leu*. *suionicum* and *Leu*. *pseudomesenteroides* were calculated using a stand-alone software (http://www.ezbiocloud.net/sw/oat)^64^, with the following recommended parameters: minimum length, 700 bp; minimum identity, 70%; minimum alignment, 50%; BLAST window size, 1000 bp; and step size, 200 bp. The pair-wise *in silico* DDH values among the whole genomes were computed using the server-based genome-to-genome distance calculator ver. 2.1 (http://ggdc.dsmz.de/distcalc2.php)^65^, with BLAST+ for genome alignments^66^. The pair-wise relatedness values of ANI and *in silico* DDH were visualized as heat-maps and hierarchical clustering using GENE-E (http://www.broadinstitute.org/cancer/software/GENE-E/).

### Pan- and core-genome analyses and a core-genome-based phylogenetic analysis

Pan- and core-genome analyses were performed using a bacterial pan-genome analysis pipeline (BPGA, ver. 1.2)^67^. The core-genome was extracted from the whole genomes of all *Leu*. *mesenteroides* strains using the USEARCH program (ver. 9.0)^68^, with a 50% sequence identity cut-off, available in BPGA. The concatenated amino acid sequences of the core-genome were aligned using the MUSCLE program (ver. 3.8.31)^69^. A core-genome-based phylogenetic tree with bootstrap values (1,000 replicates) was constructed based on a maximum likelihood algorithm using the MEGA ver. 7 software^70^.

### Relatedness based on molecular phenotypes and COG analysis

Clustering of functional genes derived from the whole genomes of *Leu*. *mesenteroides* strains was performed using the USEARCH program against the COG database within BPGA, with a default parameter setting. The clustered outputs were presented as gene presence/absence binary matrices in each genome and they were plotted using the GENE-E program with one minus the Pearson correlation distances for clustering of rows (genes) and columns (genomes).

For the functional characterization of the genomes of *Leu*. *mesenteroides* (or the core-genome and accessory/unique-genome of *Leu*. *mesenteroides*) and closely related taxa, *Leuconostoc* sp., *Fructobacillus* sp., and *Weissella* sp., functional genes derived from their respective genomes were COG-categorized using the USEARCH program and the portions of genes assigned to each COG category were expressed as relative percentages. For the functional comparison among *Leu*. *mesenteroides* and closely related taxa, average values of the relative percentages in each COG category within the taxa were used.

### KEGG analysis and reconstruction of fermentative metabolic pathways

Predicted proteins derived from the whole genomes of *Leu*. *mesenteroides* strains were submitted to BlastKOALA (http://www.kegg.jp/blastkoala/)^71^ for functional annotation based on KEGG Orthology (KO), and the metabolic and regulatory pathways of *Leu*. *mesenteroides* strains based on KO numbers were generated using the iPath v2 module (http://pathways.embl.de/iPath2.cgi#). Metabolic pathways in the KEGG pathways were displayed by line thickness and color based on the numbers of *Leu*. *mesenteroides* strains harboring genes with the same KO numbers. To investigate the fermentative metabolic features of *Leu*. *mesenteroides*, fermentative metabolic pathways of *Leu*. *mesenteroides* strains for carbohydrates were reconstructed based on the predicted KEGG pathways and EC numbers. In addition, the presence or absence of the metabolic genes in each *Leu*. *mesenteroides* strain was manually confirmed through BLASTP analyses against the genomes of *Leu*. *mesenteroides* strains, using reference protein sequences available in other closely related strains. The carbohydrate fermentation capabilities of *Leu*. *mesenteroides* strains were tested by using API 50 CH system (bioMèrieux, France) according to the manufacturer’s instructions and the type strains of four *Leu*. *mesenteroides* subspecies [ssp. *mesenteroides* ATCC 8293^T^, ssp. *jonggajibkimchii* DRC 1506^T^, ssp. *dextranicum* KACC 12315^T^ (=DSM 20484^T^), and ssp. *cremoris* KCTC 3529^T^ (=ATCC 19254^T^)] harboring different carbohydrate transport systems were used. In addition, dextran production of four *Leu*. *mesenteroides* subspecies was evaluated by mucoid properties of colonies grown on MRS agar supplemented with 5% (w/v) sucrose instead of glucose^2^.

### Expressional analysis of *Leu*. *mesenteroides* during kimchi fermentation

Kimchi metatranscriptomic sequencing data (deposited in GenBank with the acc. no. of SRX128705) that were obtained at 7, 13, 18, 25, and 29 days of kimchi fermentation in the previous study^7^ were used to investigate the metabolic features of *Leu*. *mesenteroides* during kimchi fermentation. Total mRNA sequencing reads with high quality for each kimchi sample were obtained from the raw metatranscriptomic sequencing data, as described previously^7^. The total mRNA sequencing reads of each kimchi sample were matched to the genomes of 17 *Leu*. *mesenteroides* strains and other kimchi LAB identified from the kimchi samples (*Leu*. *gasicomitatum* LMG 18811, *Leu*. *gelidum* JB7, *Leu*. *carnosum* JB16, *L. sakei* subsp. *sakei* 23 K, and *Weissella koreensis* KACC 15510) that were reported in the previous study^7^ using the BWA software^72^, based on the matching criteria of best-match with a 90% minimum identity and 20 bp minimum alignment, and putative *Leu*. *mesenteroides* sequencing reads were obtained. RPKM values (read numbers per kb of each coding sequences (CDS), per million mapped reads) for the quantification of the relative gene expressions were calculated based on *Leu*. *mesenteroides* mRNA sequencing reads that mapped onto CDS of 17 *Leu*. *mesenteroides* strains. Metabolic mapping of *Leu*. *mesenteroides* mRNA sequencing reads against the KEGG pathways of *Leu*. *mesenteroides* strains was quantitatively performed, and transcriptional expression levels of respective metabolic pathways at each kimchi fermentation time were displayed by line thickness and color change, based on the RPKM values of genes corresponding to the metabolic pathways, as described previously^73^. In addition, the transcriptional expression profiles of genes corresponding to the fermentative metabolic pathways of *Leu*. *mesenteroides* for carbohydrates during kimchi fermentation were indicated by heatmap based on their RPKM values.

## Electronic supplementary material

Supplementary material

Supplementary Table S1
