# Supplementary material for: Pan-genomic and transcriptomic analyses of Leuconostoc mesenteroides provide insights into its genomic and metabolic features and roles in kimchi fermentation
Source: Sci Rep. 2017 Sep 14;7:11504. doi: 10.1038/s41598-017-12016-z (PMC5599536; doi:10.1038/s41598-017-12016-z)
Supplement: Supplementary file 1 — Supplementary material [file 41598_2017_12016_MOESM1_ESM.pdf]

Supplementary Material

Pan-genomic and transcriptomic analyses of *Leuconostoc mesenteroides* provide insights into its genomic and metabolic features and roles in kimchi fermentation

Byung Hee Chun[1,†], Kyung Hyun Kim[1,†], Hye Hee Jeon, Se Hee Lee[2,*], and Che Ok Jeon[1,*]

[1]*Department of Life Science, Chung-Ang University, Seoul 06974, Republic of Korea*

[2]*Microbiology and Functionality Research Group, World Institute of Kimchi, Gwangju 61755, Republic of Korea*

*Corresponding author: Che Ok Jeon (cojeon@cau.ac.kr).

*Co-corresponding author: Se Hee Lee (leesehee@wikim.re.kr)

†These authors contributed equally to this study.

Supplementary Fig. S1. Heat-map based on average nucleotide identity (ANI) values (%) showing relatedness among *Leu. mesenteroides* strains and the type strains of *Leu. suionicum* (strain DSM 20241^{T}) and *Leu. pseudomesenteroides* (strain KCTC 3652^{T}) in Table 1. Strain names as described in GenBank or validated names are given as row and column labels and the type strains are highlighted in bold. Hierarchical clustering on the top is represented by a dendrogram, constructed by a simple linkage of the ANI values.

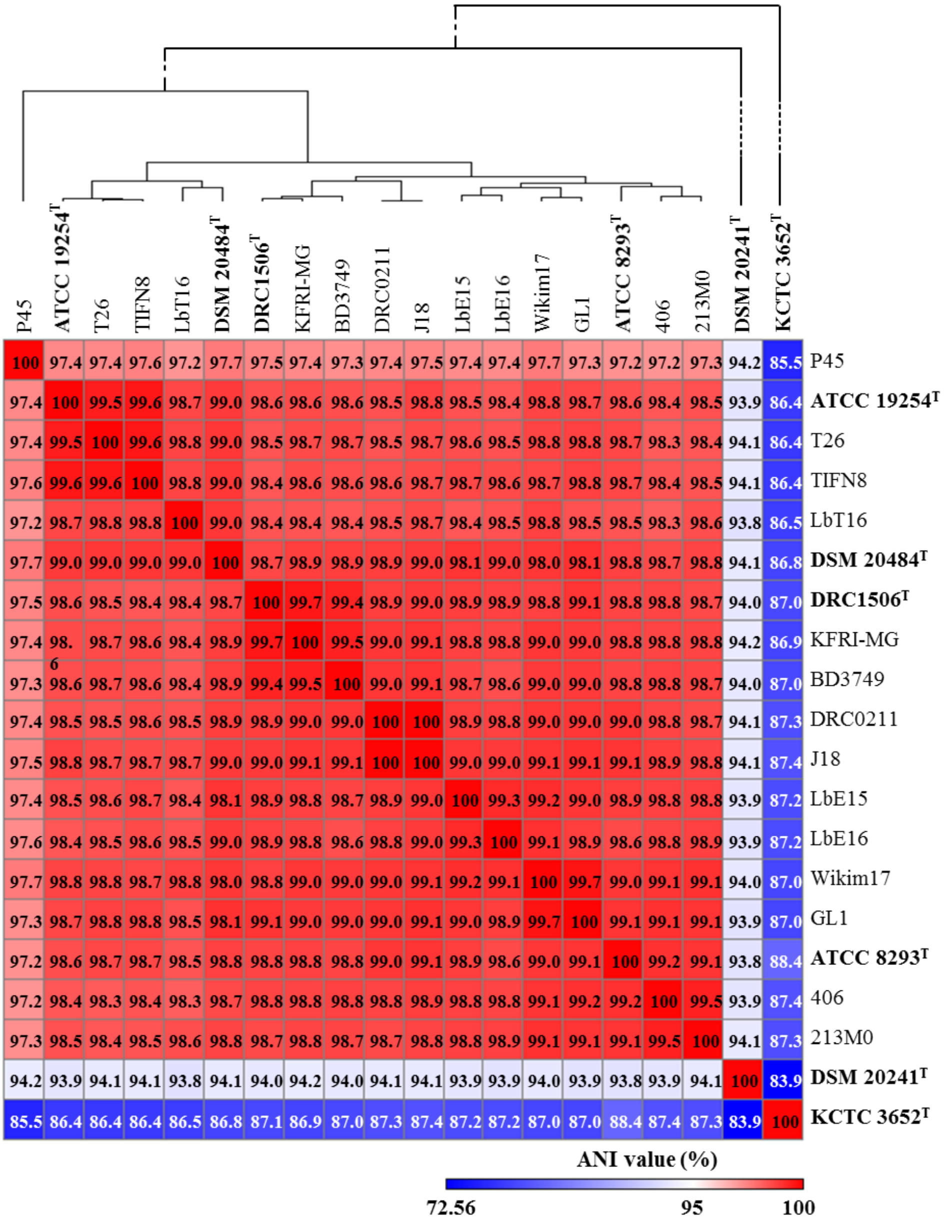

Supplementary Fig. S2. Heat-map based on *in silico* DNA-DNA hybridization (DDH) values (%) showing relatedness among *Leu. mesenteroides* strains and the type strains of *Leu. suionicum* (strain DSM 20241^{T}) and *Leu. pseudomesenteroides* (strain KCTC 3652^{T}) in Table 1. Strain names as described in GenBank or validated names are given as row and column labels and the type strains are highlighted in bold. Hierarchical clustering on the top is represented by a dendrogram, constructed by a simple linkage of *in silico* DDH values.

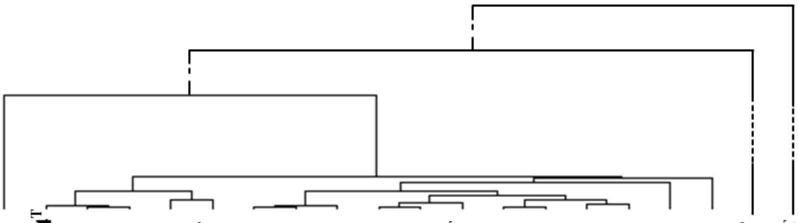

	P45	**ATCC 19254^{T}**	T26	TIFN8	LbT16	**DSM 20484^{T}**	**DRC1506^{T}**	KFRI-MG	BD3749	213M0	406	**ATCC 8293^{T}**	GL1	Wikim17	LbE15	LbE16	J18	DRC0211	**DSM 20241^{T}**	**KCTC 3652^{T}**
P45	100	78.9	79.0	79.0	77.5	79.7	79.8	79.2	79.2	79.6	78.8	79.4	79.4	80.5	78.5	78.2	80.2	80.0	57.4	47.2
**ATCC 19254^{T}**	78.9	100	96.0	97.4	91.5	91.5	88.5	89.8	89.8	89.0	89.7	90.9	91.3	90.4	88.7	88.4	89.6	89.2	56.0	50.0
T26	79.0	96.0	100	97.8	91.0	92.7	88.3	89.6	90.0	88.0	88.5	90.6	91.6	90.3	88.7	88.4	89.1	88.7	56.2	50.1
TIFN8	79.0	97.4	97.8	100	90.5	91.4	86.8	89.7	89.0	88.9	89.2	90.1	91.6	89.4	89.0	88.8	89.0	88.6	56.4	52.5
LbT16	77.5	91.5	91.0	90.5	100	93.0	88.5	89.5	88.4	89.3	89.2	89.0	90.7	90.9	87.9	88.0	90.5	90.2	55.6	51.8
**DSM 20484^{T}**	79.7	91.5	92.7	91.4	93.0	100	90.1	91.8	91.6	91.6	91.7	91.9	94.0	92.5	92.2	92.0	91.9	91.8	56.9	53.4
**DRC1506^{T}**	79.8	88.5	88.3	86.8	88.5	90.1	100	98.2	96.1	90.9	91.8	91.5	93.5	91.0	91.6	92.0	92.5	92.3	56.9	54.0
KFRI-MG	79.2	89.8	89.6	89.7	89.5	91.8	98.2	100	96.7	91.7	91.6	91.9	92.3	92.6	91.1	91.6	93.6	93.6	57.4	53.8
BD3749	79.2	89.8	90.0	89.0	88.4	91.6	96.1	96.7	100	91.3	92.1	91.8	93.5	92.4	90.8	90.3	94.2	94.1	56.6	54.1
213M0	79.6	89.0	88.0	88.9	89.3	91.6	90.9	91.7	91.3	100	97.5	94.7	94.3	93.1	91.1	91.1	91.6	91.3	56.3	55.1
406	78.8	89.7	88.5	89.2	89.2	91.7	91.8	91.6	92.1	97.5	100	95.2	94.4	94.0	90.8	91.1	92.3	91.9	55.9	56.1
**ATCC 8293^{T}**	79.4	90.9	90.6	90.1	89.0	91.9	91.5	91.9	91.8	94.7	95.2	100	94.9	93.9	92.4	91.3	93.2	93.1	55.9	59.8
GL1	79.4	91.3	91.6	91.6	90.7	94.0	93.5	92.3	93.5	94.3	94.4	94.9	100	99.1	93.3	92.7	93.8	93.7	55.9	53.4
Wikim17	80.5	90.4	90.3	89.4	90.9	92.5	91.0	92.6	92.4	93.1	94.0	93.9	99.1	100	94.6	93.6	93.0	92.8	55.7	54.4
LbE15	78.5	88.7	88.7	89.0	87.9	92.2	91.6	91.1	90.8	91.1	90.8	92.4	93.3	94.6	100	94.7	92.5	92.1	55.6	53.6
LbE16	78.2	88.4	88.4	88.8	88.0	92.0	92.0	91.6	90.3	91.1	91.1	91.3	92.7	93.6	94.7	100	92.2	91.9	55.5	53.0
J18	80.2	89.6	89.1	89.0	90.5	91.9	92.5	93.6	94.2	91.6	92.3	93.2	93.8	93.0	92.5	92.2	100	78.0	57.0	54.9
DRC0211	80.0	89.2	88.7	88.6	90.2	91.8	92.3	93.6	94.1	91.3	91.9	93.1	93.7	92.8	92.1	91.9	78.0	100	56.9	54.9
**DSM 20241^{T}**	57.4	56.0	56.2	56.4	55.6	56.9	56.9	57.4	56.6	56.3	55.9	55.9	55.9	55.7	55.6	55.5	57.0	56.9	100	40.8
**KCTC 3652^{T}**	47.2	50.0	50.1	52.5	51.8	53.4	54.0	53.8	54.1	55.1	56.1	59.8	53.4	54.4	53.6	53.0	54.9	54.9	40.8	100

***In silico* DDH value (%)**

40.8 **70** **100**

Supplementary Fig. S3. Histograms showing the distributions of the core-, accessary-, and unique-genomes in the pan-genome of *Leu. mesenteroides* strains. Genes identified from 15–16 *Leu. mesenteroides* genomes with the increase of gene numbers in the accessary genome were defined as the soft core-genome of *Leu. mesenteroides*.

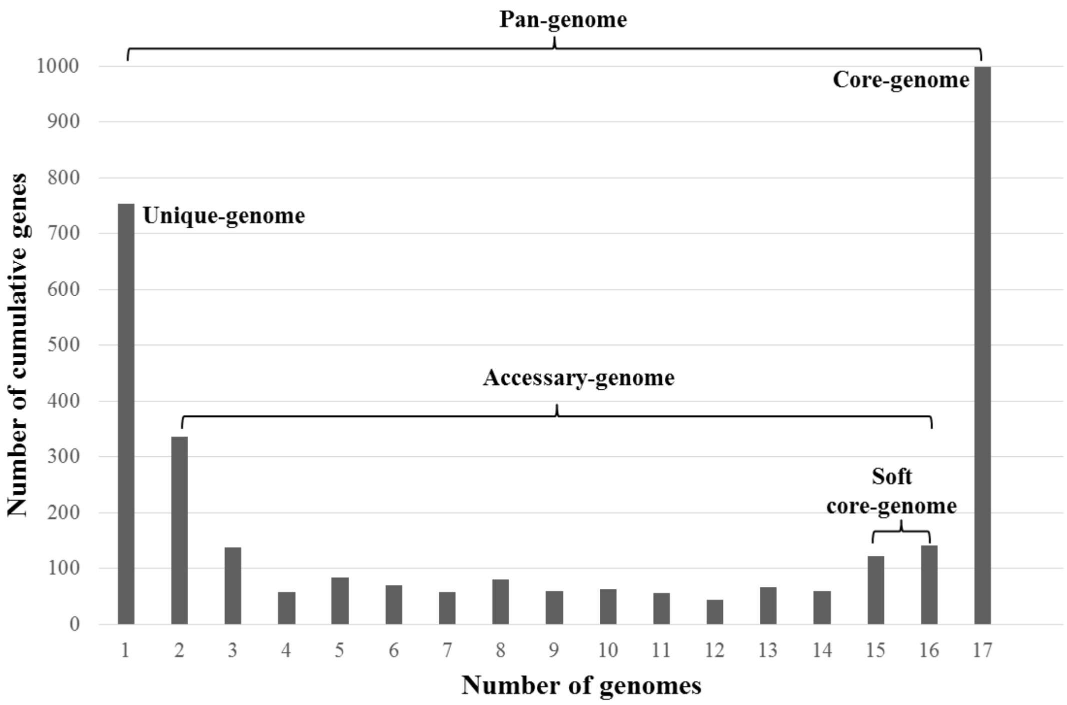

Supplementary Fig. S4. Relative abundances of *Leu. mesenteroides* mRNA sequencing reads for total LAB mRNA sequencing reads during the kimchi fermentation.

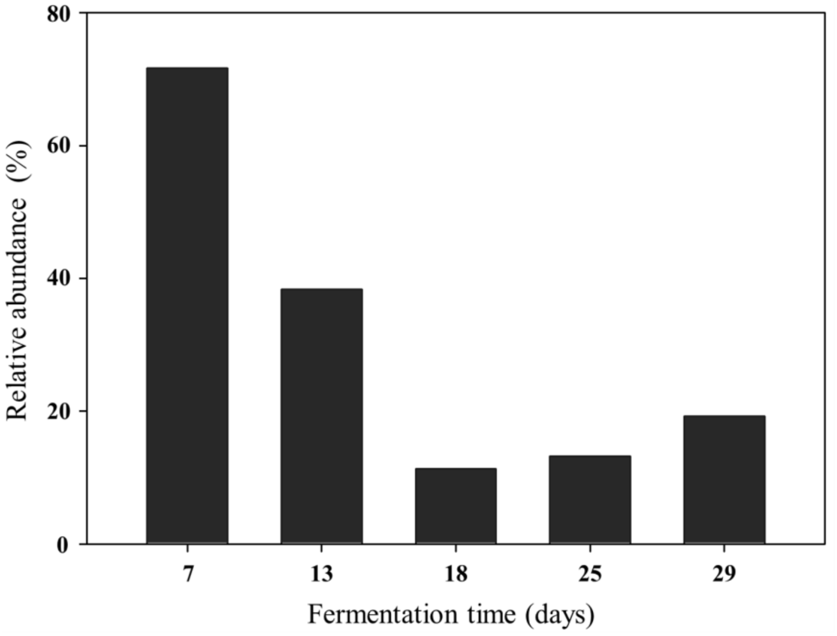

Supplementary Table S1. Lists of 999 genes of the core-genome, 1,432 genes of the accessory-genome (present in more than two strains), and 754 genes of the unique genome present in 17 *Leu. mesenteroides* strains, which are indicated in a separate EXCEL file, Supplementary Table S1.

Supplementary Table S2. List of *Leu. mesenteroides* genomes deficient for genes encoding proteins constituting the reconstructed fermentative metabolic pathways of *Leu. mesenteroides* strains.

**Protein**	**E.C number**	**Genome**
Fructose-bisphosphate aldolase	4.1.2.13	DRC1506^{T}, KFRI-MG, Wikim17, 406, GL1, ATCC 8293^{T}, J18, DRC0211, LbE16, DSM 20484^{T}, LbE15, ATCC 19254^{T}, T26, BD3749, 213M0, LbT16
Ribulokinase	2.7.1.16	DRC1506^{T}, Wikim17, 406, GL1, LbE16, DSM 20484^{T}, LbE15, ATCC 19254^{T}, LbT16, T26, BD3749, 213M0
L-Arabinose isomerase	5.3.1.4	DRC1506^{T}, Wikim17, 406, LbE16, DSM 20484^{T}, LbE15, ATCC 19254^{T}, T26, 213M0
Malate dehydrogenase	1.1.1.37	DRC1506^{T}, KFRI-MG, Wikim17, 406, GL1, J18, DRC0211, LbE16, LbE15, BD3749
Ribulose phosphate epimerase	5.1.3.4	DRC1506^{T}, Wikim17, 406, DSM 20484^{T}, ATCC 19254^{T}, T26, 213M0
PTS cellobiose transporter subunit IIA	2.7.1.205	406, LbE16, LbE15, ATCC 19254^{T}, LbT16, T26, 213M0
PTS cellobiose transporter subunit IIB	2.7.1.205	406, LbE15, ATCC 19254^{T}, LbT16, T26, 213M0
Glucosyltransferase	2.4.1.5	Wikim17, GL1, ATCC 19254^{T}, LbT16, T26
Fumarate reductase	1.3.5.4	DRC1506^{T}, Wikim17, GL1, DSM 20484^{T}, ATCC 19254^{T}, T26
Xylose isomerase	5.3.1.5	KFRI-MG, DSM 20484^{T}, ATCC 19254^{T}, LbT16, T26
Pyruvate oxidase	1.2.3.3	P45, ATCC 19254^{T}, LbT16, T26, 213M0
Maltose phosphorylase	2.4.1.8	DSM 20484^{T}, ATCC 19254^{T}, LbT16, T26
Mannose-6-phosphate isomerase	5.3.1.8	KFRI-MG, ATCC 19254^{T}, LbT16, T26
6-Phospho-β-glucosidase	3.2.1.86	LbE16, ATCC 19254^{T}, LbT16, T26
Mannitol dehydrogenase	1.1.1.67	DSM 20484^{T}, ATCC 19254^{T}, T26
PTS cellobiose transporter subunit IIC	2.7.1.205	ATCC 19254^{T}, LbT16, T26
β-Galactosidase (lacZ)	3.2.1.23	KFRI-MG, ATCC 19254^{T}
PTS sucrose transporter subunit IIABC	2.7.1.211	ATCC 19254^{T}, T26
Fructokinase	2.7.1.4	ATCC 19254^{T}, T26
Pyruvate dehydrogenase	1.2.4.1	ATCC 19254^{T}, T26
Dihydrolipoyl dehydrogenase	1.8.1.4	ATCC 19254^{T}, T26
Butanediol dehydrogenase	1.1.1.4	ATCC 19254^{T}, T26
β-Fructofuranosidase<br>Sucrose-6-phosphate hydrolase	3.2.1.26	ATCC 19254^{T}, T26
PTS system, trehalose-specific IIB/IIC component	2.7.1.201	LbE15, T26
Galactose-1-phosphate uridylyltransferase	2.7.7.12	KFRI-MG
Mannose-specific PTS system component IID	2.7.1.191	KFRI-MG
Sucrose phosphorylase	2.4.1.7	LbT16
Xylulokinase	2.7.1.17	LbT16
Triosephosphate isomerase	5.3.1.1	GL1
Enolase	4.2.1.11	GL1

Supplementary Table S3. Comparison of dextran production and fermentative metabolic properties of the type strains of four *Leu. mesenteroides* subspecies

Strains; 1. *Leu. mesenteroides* subsp. *mesenteroides* ATCC 8293^T; 2, *Leu. mesenteroides* ssp. *jonggajibkimchii* DRC1506^T; 3, *Leu. mesenteroides* subsp. *dextranicum* KACC 12315^T (=DSM 20484^T); 4, *Leu. mesenteroides* subsp. *cremoris* KCTC 3529^T (=ATCC 19254^T). All strains are positive for acid production (fermentation) from D-glucose, *N*-acetylglucosamine, arbutin, melibiose, sucrose, potassium gluconate, D-raffinose, and turanose. All strains are negative for acid production (fermentation) from glycerol, erythritol, D-arabinose, L-xylose, D-adonitol, methyl-*β*-D-xylopyranoside, L-sorbose, methyl-*α*-D-mannopyranoside, L-rhamnose, dulcitol, inositol, D-sorbitol, inulin, melezitose, starch, glycogen, xylitol, D-lyxose, D-tagatose, fucose, D-arabitol, and L-arabitol. +, positive; –, negative.

**Characteristic**	**1**	**2**	**3**	**4**
Dextran production	+	+	+	–
Fermentation (acid production) of:				
Amygdalin, L-arabinose, aesculin, D-fructose, D-mannose, D-lactose, maltose, methyl-D-glucopyranoside, salicin, trehalose, D-xylose, potassium 5-ketogluconate	+	+	+	–
Cellobiose, gentiobiose	+	–	+	–
Galactose	+	+	–	+
Potassium 2-ketogluconate	–	+	–	+
Mannitol, D-ribose	+	+	–	–
